# Amino Acids Hydrolyzed from Animal Carcasses Are a Good Additive for the Production of Bio-organic Fertilizer

**DOI:** 10.3389/fmicb.2016.01290

**Published:** 2016-08-15

**Authors:** Hongjun Liu, Dandan Chen, Ruifu Zhang, Xinnan Hang, Rong Li, Qirong Shen

**Affiliations:** ^1^Jiangsu Key Lab and Engineering Center for Solid Organic Waste Utilization, National Enginnering Research Center for Organic-based Fertilizers, Jiangsu Collaborative Innovation Center for Solid Organic Waste Resource Utilization, Nanjing Agricultural UniversityNanjing, China; ^2^Key Laboratory of Microbial Resources Collection and Preservation, Ministry of Agriculture, Institute of Agricultural Resources and Regional Planning, Chinese Academy of Agricultural SciencesBeijing, China

**Keywords:** bio-organic fertilizer, compound liquid amino acids, Illumine-MiSeq sequencing, microbial community, plant growth-promoting rhizobacteria, solid-state fermentation

## Abstract

High-quality bio-organic fertilizers (BIOs) cannot be produced without the addition of some proteins. In this study, compound liquid amino acids (CLAA) from animal carcasses were utilized as additives into matured composts to create novel BIOs containing plant growth-promoting rhizobacteria (PGPR). The results showed that adding CLAA and inoculating bacteria meanwhile resulted in failed solid-state fermentation (SSF) due to the higher H^+^ contents. While after pre-compost for 4 days before PGPR inoculation, treatments of matured chicken or pig manure added with 0.2 ml g^-1^ of CLAA resulted in a maximum biomass of functional strains. Illumine-MiSeq sequencing and Real-Time PCR results showed that the CLAA addition decreased the bacterial abundance and richness, altered the bacterial community structure and changed the relative abundance of some microbial groups. This study offers a high value-added utilization of waste protein resources for producing economical, high-quality BIO.

## Introduction

The use of large amounts of chemical fertilizer to ensure high crop yields in China has caused serious agricultural ecology and environmental issues (Silva et al., 2010; Verger and Boobis, 2013). Thus, there is a need to seek environmentally sustainable agricultural practices to complement chemical-based agriculture. Alternatively, the application of bio-organic fertilizers (BIOs) that cannot only supply plant nutrients, but also improve soil quality (Agriculture Ministry, 2004; Huang et al., 2014; Schoebitz et al., 2014), has become popular in sustainable agriculture. In general, BIOs are prepared by the solid-state fermentation (SSF) of mature compost with microbial agents (Qiu et al., 2012; Liu et al., 2014). However, due to the limitation of the available nutrients in mature composts, high-quality BIOs cannot be produced without some additional protein-containing resources, such as rapeseed meal, corn flour, soybean cake, and blue algal sludge, which have been used as additives to support the reproduction of the functional microbes (Huang et al., 2014). However, with the increasing price of rapeseed meal, corn flour, and soybean cake, the cost of produced BIOs has become unbearably higher (Zhang et al., 2014). Moreover, blue algal sludge cannot be steadily provided, limiting the development of commercial BIOs. Thus, it is desired to discover novel additive nutrients to produce BIOs.

Currently, the improvement of Chinese living standards needs the increasing development of animal husbandry (Wei et al., 2015), especially, the scale and consumption of pork has become the world’s largest industry in China (Han et al., 2011). However, the natural pig mortality rate of 10–20% in the highly intensive animal production system (Edwards, 2002) often leads to a huge amount of dead pigs in China annually (Dai et al., 2015), showing a great risk to the environment and or even to human health (Li et al., 2013). Thus, it is necessary to explore strategies to deal with the animal carcasses and maintain the development of pig husbandry. In our previous study, the sulfuric acid solution was successfully used to hydrolyze animal carcasses to create compound liquid amino acids (CLAA; China, ZL201410042218.3). Subsequently, the efficient utilization of CLAA does cause public concern. As a superior protein resource, if it could be used as protein resource in the SSF for the growth of plant growth-promoting rhizobacteria (PGPR), the problem could be resolved clearly and the costs of BIOs can be notably decreased meanwhile.

The pivotal role of SSF in preparing BIOs is to promote the microbial agent predominating in the mature compost, which usually contains the complex indigenous microbial composition (Dukare et al., 2011). Additive protein resources cannot only enhance the multiplication of the microbial agent, but can also stimulate the growth of indigenous microbes (Kato and Miura, 2008). Thus, the process of successfully reproducing the functional strain results in a high-quality BIOs associated with the complex variation of the whole microflora (Liu et al., 2014). Detailed research of the latter has still been ignored, but this may help to understand the mechanisms of the successful colonization of the functional strain. Recently, pyrosequencing of 16S rRNA and internal transcribed spacer (ITS) gene fragments has been applied for the in-depth analysis of microbial communities (MacLean et al., 2009; Hartmann et al., 2015). This method could provide an unprecedented opportunity to achieve a high throughput and deeper insight into the alterations of microbial communities during SSF.

The CLAA produced from animal carcasses using a hydrolysis process with sulfuric acid solution contained huge amounts of protein and high H^+^ concentration. This resource was first investigated for exploring BIO of PGPR in this study. The objectives of this work were to evaluate whether the high H^+^ concentration could influence the SSF process, to explore an efficient SSF technology based on CLAA to create a novel low-cost, high-quality BIO and to monitor the microflora alterations associated with the new SSF process.

## Materials and Methods

### Materials

The CLAA used as the additional nutrient resource for preparing BIO was provided by Lianye Biotechnology Co., Ltd., Jiangsu, China. It contained an H^+^ concentration of 5.23 mol L^-1^ and total nitrogen (N), total phosphorus (P) and total potassium (K) contents of 41.23, 0.16, and 0.39 g L^-1^, respectively, with a free amino acid concentration of 124.16 g L^-1^. Matured chicken manure compost was provided by Huinong Biotechnology Co., Ltd., Jiangsu, China. This had pH, moisture, total carbon, total nitrogen (N), phosphorus (P), and potassium (K) contents of 8.34, 28.37%, 144.8 g kg^-1^, 13.4 g kg^-1^, 8.47g kg^-1^, and 10.2 g kg^-1^, respectively. Matured pig manure compost was provided by Aboluo Biotechnology Co., Ltd., Jiangsu, China. It had moisture, total carbon, total nitrogen (N), phosphorus (P), and potassium (K) contents of 7.32, 26.23%, 194.8 g kg^-1^, 17.6 g kg^-1^, 9.26 g kg^-1^, and 15.5 g kg^-1^, respectively. The bacterial strains *Bacillus amyloliquefaciens* SQR9 (Cao et al., 2011), *B. amyloliquefaciens* NJN-6 (Ling et al., 2012) and *Paenibacillus polymyxa* SQR21-*gfp* (Wang et al., 2013) were previously isolated in our lab and identified to be the biocontrol agents for suppressing Fusarium wilts of cucumber, watermelon and banana, respectively.

### Experimental Design

The cells of SQR9, NJN-6, and SQR21-*gfp* were pre-cultured in liquid LB medium at 170 rpm and 30°C for 24 h, then, harvested by centrifugation at 6000 rpm for 5 min, washed three times with sterilized water, and suspended in the same volume of sterilized water.

To determine the best additive concentration of CLAA for SSF using strain SQR9, matured chicken manure compost containing five different concentrations of CLAA [0.05 ml g^-1^ (CM 0.05), 0.1 ml g^-1^ (CM 0.1), 0.2 ml g^-1^ (CM 0.2), 0.3 ml g^-1^ (CM 0.3) and 0.4 ml g^-1^ (CM 0.4)] (DW) and a control (CMCK) without CLAA added were arranged in small, cone-shaped windrows (15 cm high, 30 cm diameter base, approximately 3 kg DW each). At the same time, matured pig manure compost containing four different concentrations of CLAA, 0.15 ml g^-1^ (PM0.15), 0.2 ml g^-1^ (PM 0.2), 0.25 ml g^-1^ (PM 0.25), and 0.3 ml g^-1^ (PM 0.3), and a control (PMCK) without CLAA added were also arranged in a similar manner. Because the high H^+^ concentration could influence the SSF process, strain SQR9 was inoculated into the mixtures in the beginning or after 6 days of pre-composting in the treatments and control, the pH of which were detected daily. The initial inoculation size of strain SQR9 was approximately 5 × 10^7^ CFU g^-1^ DW, and the moisture content of the mixtures was maintained at 40–45%. The mixtures were maintained at room temperature (20–30°C) for 6 days and manually turned on a daily basis to promote the growth of the inoculated PGPR strain.

To investigate the optimal time for the pre-compost, SQR9 cells were inoculated after 0, 2, 4, and 6 days pre-compost in matured chicken (CMP0, CMP2, CMP4, and CMP6) and pig manure (PMP0, PMP2, PMP4, and PMP6), respectively, with 0.2 ml g^-1^ of the CLAA. The culture conditions were the same as described above.

Finally, 4 days pre-compost and the additive amount of 0.2 ml g^-1^ CLAA were chosen for the SSF of other two bacteria NJN-6 and SQR21-gfp (PCNJN-6 and PCSQR21-gfp). The treatments without CLAA were considered as the controls (CKNJN-6 and CKSQR21-*gfp*). The numbers of cells of SQR21-*gfp*, SQR9, and NJN-6 were determined according to Zhang et al. (2014).

### Scaled up SSF

For the scaled up SSF, matured chicken manure with 0.2 ml g^-1^ of CLAA, was arranged in small, cone-shaped windrows (50 kg DW) and maintained at room temperature (20–30°C) for 4 days of pre-compost. Then, the mixtures were inoculated with strain SQR9 for the next 4 days of SSF. After the turning of the piles (once daily), four sub-samples were taken from symmetrical locations around the heap and combined to form a composite sample at three points during SFF as follows: matured chicken manure treated as the control (CK), mixed piles of matured chicken manure and CLAA pre-composted for 4 days (PC), and matured chicken manure with or without CLAA inoculated with SQR9 after SSF for 4 days, named PCBIO and CKBIO, respectively. Triplicate composite samples of each treatment were collected and stored at 4°C immediately prior to analysis. The physicochemical properties of the fertilizer samples were detected according to Zhang et al. (2014).

### DNA Extraction

Total fertilizer DNA was extracted using UltraClean Soil DNA Isolation Kits (Mo Bio Laboratories Inc., Carlsbad, CA, USA) according to the manufacturer’s protocol. The concentration and quality of the DNA were determined using a spectrophotometer (NanoDrop 2000, USA). Then, the total numbers of bacteria and fungi were quantified by Real-Time PCR (qPCR) according to Shen et al. (2013). Each sample was performed in three replicates, and the results were expressed as log (copies g^-1^) dry soil.

### MiSeq Sequencing

The V4 hypervariable regions of the 16S rRNA gene using primers 520F (5′-AYTGGGYDTAAAGNG-3′) and 802R (5′-TACNVGGGTATCTAATCC-3′) (Claesson et al., 2009) and the ITS region employing primers ITS1F (5′-CTTGGTCATTTAGAGGAAGTAA-3′) and ITS2 (5′-GCTGCGTTCTTCATCGATGC-3′) (Huang X.Q. et al., 2015) were amplified for bacteria and fungi, respectively. The unique 6-nt barcodes attached to the reverse primer used to distinguish each sample are showed in Supplementary Table S1. The programs of amplification and pyrosequencing of the bacterial 16S rRNA and fungal ITS sequences were performed at Personal Biotechnology Co., Ltd. (Shanghai, China) on the Illumina MiSeq instrument (USA). All sequences were deposited in the NCBI Sequence Read Archive (SRA) database (accession number SRP066872).

### Pyrosequencing Data Processing

Sequences were processed, quality controlled, and annotated according to Huang X.Q. et al. (2015). Then, 21,099 sequences per sample of the 16S rRNA genes for bacterial analysis and 6,275 sequences per sample of the ITS sequences for fungal analysis were randomly selected. Richness and diversity were calculated by MOTHUR with an OTU cut-off of 0.03 (Shen et al., 2015). To compare the bacterial and fungal microbial community structure among all the fertilizer samples, principal coordinate analysis (PCoA) based on the Bray–Curtis distance metric was performed by MOTHUR. In addition, to better understand the bacterial and fungal community composition, relative abundances at genus level were compared. Finally, to examine the relationship between the analyzed bacterial genera [significant (*P* < 0.05) difference between different treatments], samples and selected environmental variables, a redundancy analysis (RDA) was carried out using CANOCO for Windows (Etten, 2005).

### Statistical Analysis

The differences among the treatments were analyzed using a one-way ANOVA, and the calculated means were subjected to Duncan’s multiple range test at *P* ≤ 0.05. SPSS v 18.0 was used for the statistical analysis (SPSS Inc., Chicago, IL, USA).

## Results

### SSF Efficiency

**FIGURE 1:**
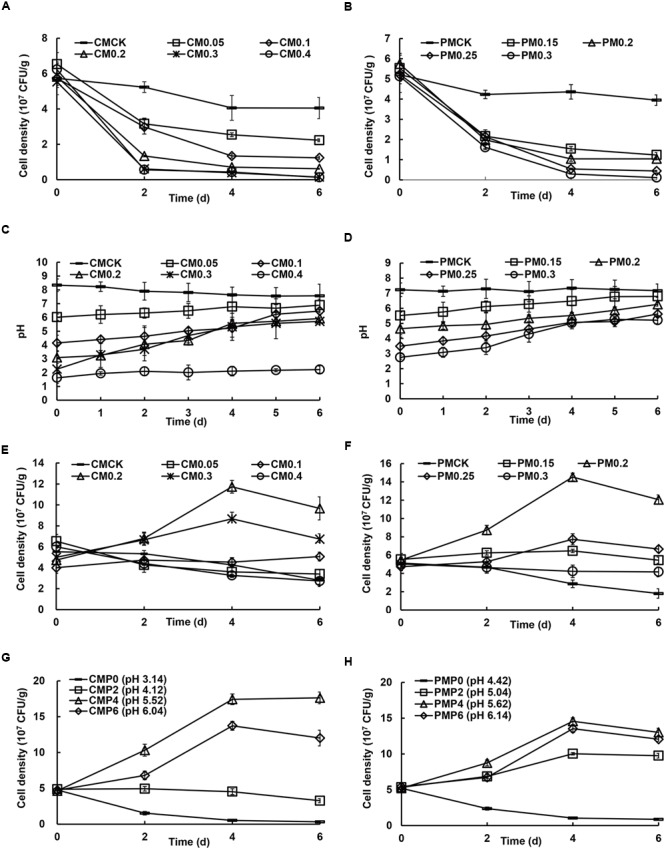
**Effects of different additive concentrations of compound liquid amino acid (CLAA) added to matured chicken **(A, C, E, and G)** and pig manure **(B, D, F, and H)** on the cell density of plant growth-promoting rhizobacteria (PGPR) strain SQR9 and pH value during solid-state fermentation (SSF) with or without pre-compost.** (**A** and **B**): cell density of SQR9 without pre-compost; (**C** and **D**): variation of pH value during pre-compost; (**E** and **F**): cell density of SQR9 with pre-compost for 6 days; (**G** and **H**): cell density of SQR9 with different initial pH value for inoculation after pre-compost for different times. CMCK, CM0.05, CM0.1, CM0.2, CM0.3, and CM0.4: addition of 0, 0.05, 0.1, 0.2, 0.3, and 0.4 ml/g of CLAA in matured chicken manure (CM), respectively; PMCK, PM0.15, PM0.2, PM0.25, and PM0.3: addition of 0, 0.15, 0.2, 0.25, and 0.3 ml/g of CLAA in matured pig manure (PM), respectively; CMP0, CMP2, CMP4, and CMP6: 0, 2, 4, and 6 days for pre-compost in matured chicken manure (CM), respectively; PMP0, PMP2. PMP4, and PMP6: 0, 2, 4, and 6 days for pre-compost in matured pig manure (PM).

Due to the CLAA addition, the H^+^ contents of all treatments (added CLAA and inoculated bacteria meanwhile) were all higher than CK, resulting in the failed SSF processes, in which the SQR9 cell density in all treatments and the control followed the same trend and decreased with the increase of CLAA contents, regardless of the use of matured chicken or pig manure composts (**Figures 1A,B**).

After 6 days pre-compost, the pH value of all treatments increased to 6–7 except the CM 0.4 treatment (**Figures 1C,D**). As shown in **Figures 1E,F**, at the 4th day, the cell density of strain SQR9 in both CM 0.2 and PM 0.2 was greater than 1 × 10^8^ CFU/g, significantly higher than that in the other treatments and the control. In addition, pre-compost for 4 days showed a higher cell density of strain SQR9 in CMP4 and PMP4 (matured chicken and matured pig manure with 0.2 ml g^-1^ of CLAA added; **Figures 1G,H**), indicating that 4 days of pre-compost was the optimal time.

Moreover, as shown in **Supplementary Figure S1**, compared to the control, the novel SSF facilitated significant growth of *B. amyloliquefaciens* NJN-6 and *P. polymyxa* SQR21-*gfp*. Therefore, the results showed that 20% CLAA (DW) is the best additive concentration for the novel SSF and should be added for 4 days before the inoculation of functional microbes.

### Physicochemical Properties of Fertilizer Samples Collected from the Enlarged SSF

Chicken manure compost with 0.2 ml g^-1^ of CLAA added was selected for the enlarged SSF experiment. The variations in different physicochemical properties of the enlarged process are shown in **Table 1**. After pre-compost, significantly higher TN content and lower pH valued were observed in PC than in CK, whereas no significant differences for the TP and TK contents were shown. At the end of the SSF, compared to CKBIO, PCBIO showed a significantly higher value of TN, indicating that the CLAA addition enriched the nitrogen nutrition in the product.

**Table 1:** Physicochemical properties of the different treatments and control.

	pH	TN (%)	TP (%)	TK (%)
CK	8.23 ± 0.12^a^	1.10 ± 0.02^c^	1.34 ± 0.02^a^	1.26 ± 0.03^a^
CKBIO	8.13 ± 0.14^a^	1.03 ± 0.01^d^	1.34 ± 0.07^a^	1.32 ± 0.05^a^
PC	5.44 ± 0.13^b^	1.59 ± 0.04^a^	1.28 ± 0.03^a^	1.32 ± 0.03^a^
PCBIO	5.66 ± 0.18^b^	1.33 ± 0.06^b^	1.37 ± 0.06^a^	1.29 ± 0.04^a^

*CK: the mature chicken manure compost; CKBIO: the mature chicken manure compost with strain SQR9 inoculation; PC: pre-compost of mixture piles of mature chicken manure and CLAA; PCBIO: inoculation of strain SQR9 in mixture piles of mature chicken manure and CLAA after pre-compost.* The values are the means with one standard error of the mean in parentheses. The different letters indicate statistically significant differences at the 0.05 probability level, according to Fisher’s least significant difference test (LSD) and Duncan’s test.

### Total Bacterial and Fungal Abundances

**FIGURE 2:**
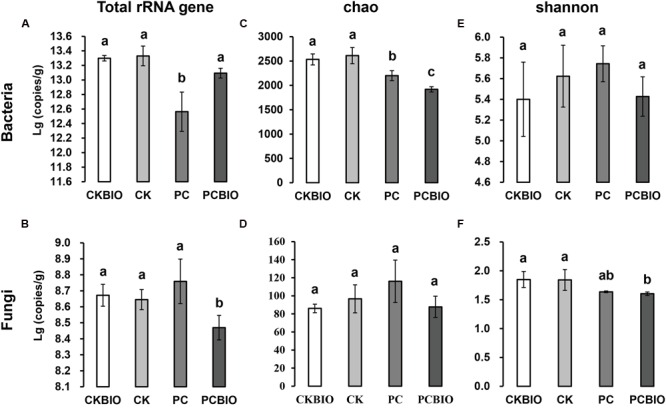
**Microbial population and community richness and diversity for different treatments and control.** Bars with different letters indicate significant differences among the four treatments, as defined by Duncan’s test (*P* < 0.05). **(A)** Total number of bacteria quantified by qPCR; **(B)** total number of fungi quantified by qPCR; **(C)** bacterial Chao index; **(D)** fungal Chao index; **(E)**: bacterial Shannon index; **(F)**: fungal Shannon index. CK: the mature chicken manure compost; CKBIO: the mature chicken manure compost with strain SQR9 inoculation; PC: pre-compost of mixture piles of mature chicken manure and CLAA; PCBIO: inoculation of strain SQR9 in mixture piles of mature chicken manure and CLAA after pre-compost.

The qPCR results showed that after CLAA addition, pre-compost significantly decreased the total bacterial abundance, while at the end of SSF, the value rose again and showed no significant difference between the two products (**Figure 2A**). For fungi, no significant difference was observed after CLAA addition, and the novel BIO produced by CLAA addition showed significantly lower abundance compared to the product from CKBIO (**Figure 2B**).

### General Analyses of the Sequencing Data

As shown in Supplementary Table S2, after quality control, a total of 726,661 bacterial sequence reads and 170,237 fungal sequence reads were obtained from 12 samples. The number of high-quality sequences per sample varied from 21,099 to 91,327 for bacteria and 6,275 to 26,045 for fungi.

### Microbial Community Richness and Diversity

The richness (Chao) and diversity (Shannon) of bacteria and fungi in different treatments are shown in **Figures 2C–F**. After pre-compost, the addition of CLAA significantly decreased the bacterial richness (Chao), while no significant differences of bacterial diversity (Shannon), fungal richness (Chao), and fungal diversity (Shannon) were observed. Significantly lower bacterial richness (Chao) and fungal diversity (Shannon) were observed in PCBIO than in the product CKBIO.

### Microbial Community Structure

**FIGURE 3:**
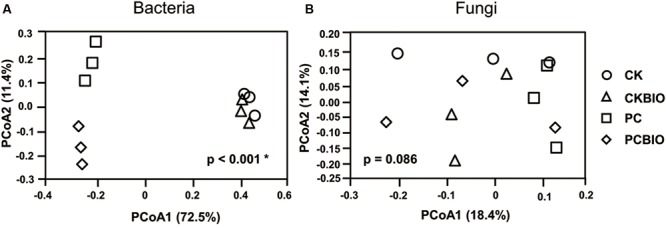
**Bacterial **(A)** and Fungal **(B)** community structure for different treatments and control.** CK: the mature chicken manure compost; CKBIO: the mature chicken manure compost with strain SQR9 inoculation; PC: pre-compost of mixture piles of mature chicken manure and CLAA; PCBIO: inoculation of strain SQR9 in mixture piles of mature chicken manure and CLAA after pre-compost.

Principal coordinates analysis based on the Bray–Curtis distance metric clearly (*p* < 0.001^∗^) showed variations in the bacterial community among the samples collected from novel SSF and the control along the first component (PCoA1; **Figure 3A**). The first two principal components could explain 73.9% of the variation of the individual samples of the total bacterial community, and the bacterial community in PCBIO was well-separated from samples collected after pre-compost along the second component (PCoA2). Nevertheless, all the samples were hardly distinguished (*p* = 0.086) from each other in the fungal community (**Figure 3B**).

### Microbial Community Composition

**FIGURE 4:**
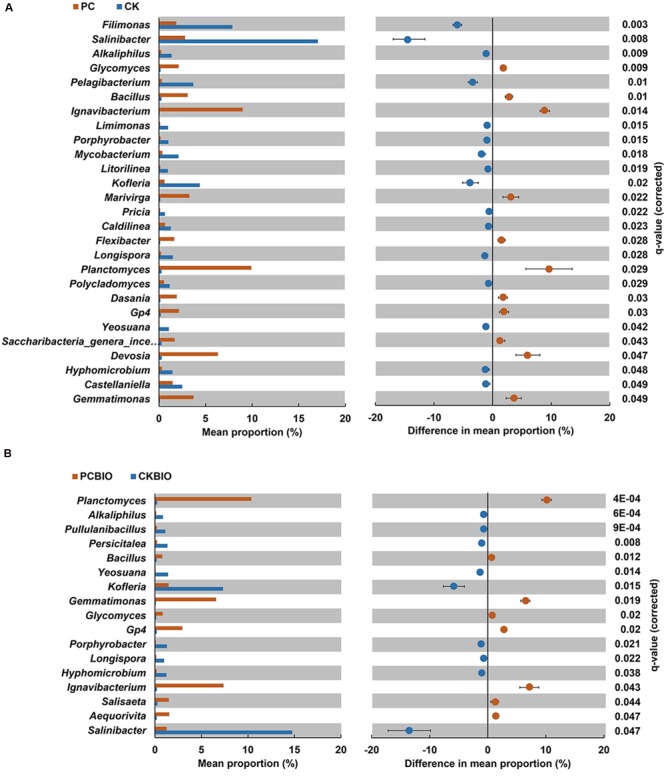
**Bacterial and fungal genera (relative abundance > 0.1%) analyzed with *P*-values adjusted using the Benjamini-Hochberg method at *P* < 0.05 among PC compared with CK **(A)** and PCBIO compared with CKBIO **(B)**.** CK: the mature chicken manure compost; CKBIO: the mature chicken manure compost with strain SQR9 inoculation; PC: pre-compost of mixture piles of mature chicken manure and CLAA; PCBIO: inoculation of strain SQR9 in mixture piles of mature chicken manure and CLAA after pre-compost.

At the genus level, the bacterial and fungal genera (relative abundance > 0.1%) were analyzed with *P*-values adjusted using the Benjamini–Hochberg method at *P* < 0.05. Through the comparison between CKBIO and CK, PC and CK, PCBIO and CKBIO, only bacterial or fungal genera showing significant differences are shown in **Figure 4**.

For bacteria, no significant difference between CKBIO and CK was observed. However, compared to CK, PC significantly (*P* < 0.05) increased the relative abundance of *Bacillus*. Similarly, PCBIO showed a significantly greater relative abundance of *Bacillus* than CKBIO.

Moreover, PC significantly (*P* < 0.05) increased the relative abundance of *Planctomyces, Ignavibacterium, Devosia, Gemma timonas, Marivirga, Glycomyces, Gp4, Dasania, Saccharibacteria_genera_incertae_sedis, and Flexibacter* and reduced the relative abundance of *Salinibacter, Filimonas, Kofleria, Pelagiba cterium, Mycobacterium, Longisporum, Hyphomicrobium, Alkaliphilus, Castellaniella, Yeosuana, Limimonas, Porphyrobacter, Litorilinea, Caldilinea, Polycladomyces*, and *Pricia*, compared to CK (**Figure 4A**). PCBIO significantly (*P* < 0.05) increased *Planctomyces, Ignavibacterium, Gemmatimonas, Gp4, Aequorivita, Salisaeta*, and *Glycomyces* and reduced *Alkaliphilus, Longisporum, Pullulanibacillus, Hyphomicrobium, Persicitalea, Porphyrobacter, Yeosuana, Kofleria*, and *Salinibacter* (**Figure 4B**) compared to CKBIO. Compared to CK, the CLAA-containing fertilizers (PC and PCBIO) showed higher relative abundance of *Planctomyces, Ignavibacterium, Devosia, Bacillus, Gemmatimonas, Marivirga, Glycomyces, Gp4, Dasania, Saccharibacteria*_genera_incertae_sedis and *Flexibacter*, and lower relative abundance of *Salinibacter, Filim onas, Kofleria, Pelagibacterium, Mycobacterium, Longisporum, Hyphomicrobium, Alkaliphilus, Castellaniella, Yeosuana, Limimonas, Porphyrobacter, Litorilinea, Caldilinea, Polycla domyces*, and *Pricia*. Regardless of the effect of CLAA, the inoculation of SQR9 increased *Salisaeta, Aequorivita*, and *Glycomyces* and decreased *Pullulanibacillus, Alkaliphilus*, and *Persicitalea* compared to CK.

For fungi, no significant difference in the relative abundance of the genera was observed.

### Relationship between Selected Fertilizer Properties and Analyzed Bacterial Genera for Fertilizer Samples

**FIGURE 5:**
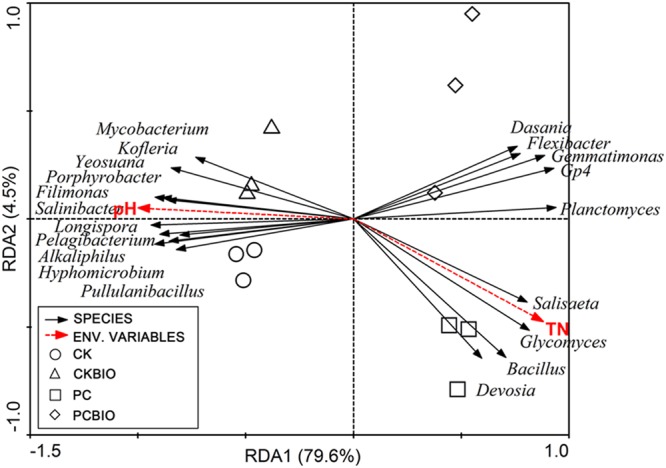
**Redundancy analysis (RDA) of the analyzed bacterial genera and selected soil properties for soil samples from different treatments and control.** CK: the mature chicken manure compost; CKBIO: the mature chicken manure compost with strain SQR9 inoculation; PC: pre-compost of mixture piles of mature chicken manure and CLAA; PCBIO: inoculation of strain SQR9 in mixture piles of mature chicken manure and CLAA after pre-compost.

Monte Carlo tests based on the selected soil chemical properties and the abundances of analyzed bacterial genera [significant (*P* < 0.05) difference between different treatments] revealed that the selected soil chemical properties were significantly correlated to variations in the analyzed bacterial genera (*p* = 0.002). RDA analysis showed that the first and second RDA components explained 84.1% of the total bacterial variations (**Figure 5**). The first component (RDA1), which explained 79.6% of the variation, separated the no CLAA-containing (CK and CKBIO) and CLAA-containing (PC and PCBIO) treatments. Moreover, the CLAA-containing (PC and PCBIO) treatments were dominated by *Dasania, Flexibater, Gemmatimonas, Gp4, Planctomycetes, Salisaeta, Glycomyces, Bacillus*, and *Devosia* and were positively related to TN but negatively to pH. Additionally, the second component (RDA2), which mainly separated the PC and PCBIO treatments, explained 4.5% of the variation.

## Discussion

Negative effects of the different additive-concentrations of CLAA on the cell density of strain SQR9 were observed when added CLAA and inoculated bacteria meanwhile. This may be due to the high H^+^ concentration in the mixture, which resulted in low initial pH and limited the microbial activity (Partanen et al., 2010). Thus the pre-compost process was subsequently carried out to explore the efficient SSF, during which the pH value increased to 5.5–6 in treatments added with less than 30 and 20% of CLAA in matured chicken and pig manure composts, respectively, and this may be due to bio-H_2_ production by the microbes (Lee et al., 2009). After pre-compost, the functional microbe was inoculated, and its numbers were increased significantly in the mixtures containing 20% of CLAA regardless of matured chicken or pig manure compost was used. Thus, the optimum amount of CLAA to add into the compost was 20%, and the recommended pre-compost time was 4 days. The results were similar to other studies, in which more or less additive of protein did not feed back to higher amounts of functional microbes (Zhang et al., 2014). In our study, more CLAA added to the mixture induced high H^+^ concentration, subsequently inhibiting the growth of functional microbes. Moreover, two bacterial strains, *B. amyloliquefaciens* NJN-6 and *P. polymyxa* SQR21, could also grow well in the novel SSF, indicating that the novel SFF is suitable for other bacteria. From the qPCR results, additional CLAA significantly decreased the bacterial abundance, possibly due to the low pH discussed above. Moreover, the inoculation of SQR9 increased the abundance of bacteria and decreased the abundance of fungi. The reason may be that the presence of non-antagonistic bacteria in a community suppressed fungal growth (De Boer et al., 2007).

Due to the high H^+^ concentration and high nitrogen content of CLAA compared to the CK and CKBIO treatments, the added CLAA treatments (PC and PCBIO) significantly decreased the fertilizer pH value and increased the fertilizer TN. Similar to our previous results, several studies have already shown that some wastes, such as blue algal sludge, rapeseed meal and soybean cake, could not only be added as protein sources to promote the growth of functional microorganisms, but also improve the nutritional contents of the produced BIOs (Huang Y. et al., 2015).

For bacteria, the total number of OTUs and the Chao and Shannon indices of CK and CKBIO were all higher than that in the PC and PCBIO treatments. This may be due to the H^+^ addition of the CLAA, which suppressed the growth of microorganisms (Sundberg et al., 2004). Moreover, the species richness of the PCBIO treatment also decreased. This could be due to the competition for nutrition between the PGPR and indigenous microorganisms (Liu et al., 2012). For fungi, no significant difference was found in the OTU numbers and Chao index among all treatments, whereas the novel-produced BIO showed the lowest value of the Shannon index, indicating that PGPR may be important contributors to BIO suppressiveness and fungistasis in a community context (Shen et al., 2013). Moreover, the rarefaction curves at 3% dissimilarity also revealed that the addition of CLAA reduced bacterial OTU numbers, while no obvious effect was observed for fungi (**Supplementary Figure S2**).

As a nutritional additive, the CLAA greatly influenced the bacterial community structure and slightly affected the fungal community structure for PCBIO products. For bacteria, the β-diversity clearly demonstrated that there was a significant fraction of variation in community diversity, which could be attributed to CLAA addition (mainly by PCoA1). These results were consistent with the previous studies that showed that concentrated monosodium glutamate wastewater as a nutrient additive could alter the bacterial community structure in BIO (Liu et al., 2014). Comparing the effect of inoculation with and without CLAA, no significant difference was observed between CKBIO and CK, but the community structure of PCBIO was shown to be significantly different from PC, indicated that with the CLAA addition, the inoculation can change the bacterial community structure of native-born microflora. In other word, *B. amyloliquefaciens* SQR9 could successfully colonize in the matured compost, due to the addition of CLAA. However, for fungi, the slightly affected community structure explained the finding of Sundberg et al. (2004) that fungi were generally more tolerant to acids than bacteria.

Microbial composition analysis revealed that no significant difference in fungal genera (relative abundance > 0.1%) was observed. However, for bacteria, although no significant difference between the CK and CKBIO treatments was observed, significant differences in the genera (relative abundance > 0.1%) levels among CK compared with PC, PC compared with PCBIO and CKBIO compared with PCBIO were observed. These results indicated that the novel SSF greatly influenced the bacterial composition.

Our RDA analysis revealed that the top bacterial and fungal genera in the CLAA-containing (PC and PCBIO) treatments were dominated by *Dasania, Flexibater, Gemmatimonas, Gp4, Planctomycetes, Salisaeta, Glycomyces, Bacillus*, and *Devosia* and were positively related to TN, but negatively to pH. This finding suggested that *Bacillus* was better able to stand lower pH than others, which roughly corresponded to the results of previous studies that the buffering capacity of *B. subtilis* cells extends to pH conditions as low as pH 2 (Fein et al., 2005). In addition, PC and PCBIO, with the higher TN, resulted in the higher relative abundance of the genus *Bacillus*, indicating that the CLAA as the nitrogen resource was suitable for the growth of the PGPR. The results were roughly in agreement with many previous studies showing that rapeseed meal (Shen et al., 2013), concentrated monosodium glutamate wastewater (Liu et al., 2014) and algal sludge (Zhang et al., 2014) were effectively utilized to create novel PGPR-containing BIOs. Additionally, the second component (RDA2) mainly separated the PC and PCBIO treatments, and explained 4.5% of the variation, indicating that the inoculated PGPR SQR9 further altered the microbial community structure in the produced BIO.

## Conclusion

The additional CLAA decreased the pH value and increased the TN content of matured chicken manure, thereby, reduced the bacterial richness and increased the relative abundance of *Bacillus* by creating a suitable environment for SQR9 colonization, which greatly influenced the bacterial community of the PCBIO products. Our work provides an efficient way to address CLAA, which not only sought a cheap medium for producing low-cost, high-quality BIO, but also guarantees sustainable development of animal husbandry.

## Author Contributions

HL collected samples, conducted lab works and wrote the manuscript. RL planned this study and revised the manuscript. RZ revised the manuscript. DC and XH joined in lab work and laboratory analyses. All authors reviewed the manuscript.

## Conflict of Interest Statement

The authors declare that the research was conducted in the absence of any commercial or financial relationships that could be construed as a potential conflict of interest.

## Supplementary Material

FIGURE S1 **Effects of the novel solid-state fermentation (SSF) on the cell density variations of plant growth-promoting rhizobacteria (PGPR) NJN-6 and SQR21-gfp**.

FIGURE S2 **Rarefaction curves of bacterial 16S rRNA genes **(A)** and fungal ITS sequences **(B)** at 97% similarity levels of the different treatments and control.** CK: the mature chicken manure compost; CKBIO: the mature chicken manure compost with strain SQR9 inoculation; PC: pre-compost of mixture piles of mature chicken manure and CLAA; PCBIO: inoculation of strain SQR9 in mixture piles of mature chicken manure and CLAA after pre-compost.
