# Supplementary material for: Amino Acids Hydrolyzed from Animal Carcasses Are a Good Additive for the Production of Bio-organic Fertilizer
Source: Front Microbiol. 2016 Aug 15;7:1290. doi: 10.3389/fmicb.2016.01290 (PMC4983570; doi:10.3389/fmicb.2016.01290)
Supplement: Supplementary file 1 [file Table_1.DOC]

**Table S1**

Barcodes of the different treatments and control used in this study. CK: the mature chicken manure compost; CKBIO: the mature chicken manure compost with strain SQR9 inoculation; PC: pre-compost of mixture piles of mature chicken manure and CLAA; PCBIO: inoculation of strain SQR9 in mixture piles of mature chicken manure and CLAA after pre-compost.

| Name | Sequence | | Sample name |
| --- | --- | --- | --- |
|  | Bacterial 16S rRNA | Fungal ITS |  |
| barcode | GCACCTA | ACACAGT | CK1 |
| barcode | CCACGAG | ACAGTCA | CK2 |
| barcode | GCACGCT | ACTCTGA | CK3 |
| barcode | GCTACGT | CATCAGA | PC1 |
| barcode | CCTACTA | CATGTCT | PC2 |
| barcode | GCTAGAG | CACAAGT | PC3 |
| barcode | ACCAACA | CTTGAGT | CKBIO1 |
| barcode | ACCAACT | CTCACGA | CKBIO2 |
| barcode | ACCAATC | CTCAGAC | CKBIO3 |
| barcode | AGTCGTC | GACTCAC | PCBIO1 |
| barcode | AGTCTGC | GAGTCGT | PCBIO2 |
| barcode | ATACAGT | GTACAGA | PCBIO3 |
