# Supplementary material for: Amino Acids Hydrolyzed from Animal Carcasses Are a Good Additive for the Production of Bio-organic Fertilizer
Source: Front Microbiol. 2016 Aug 15;7:1290. doi: 10.3389/fmicb.2016.01290 (PMC4983570; doi:10.3389/fmicb.2016.01290)
Supplement: Supplementary file 2 [file Table_2.DOC]

**Table S2** Retained sequences and OTUs that were used for further analysis after removing short, ambiguous, and low-quality reads of the different treatments and control. CK: the mature chicken manure compost; CKBIO: the mature chicken manure compost with strain SQR9 inoculation; PC: pre-compost of mixture piles of mature chicken manure and CLAA; PCBIO: inoculation of strain SQR9 in mixture piles of mature chicken manure and CLAA after pre-compost.

| Sample | Retained sequences | |
| --- | --- | --- |
|  | Bacterial 16S sequences  (3437 OTUs) | Fungal ITS sequences  (138 OTUS) |
| CKBIO1 | 75,206 | 7,751 |
| CKBIO2 | 53,857 | 6,275 |
| CKBIO3 | 70,706 | 6,661 |
| CK1 | 75,149 | 26,045 |
| CK2 | 51,081 | 17,896 |
| CK3 | 91,327 | 15,694 |
| PC1 | 67,075 | 9,159 |
| PC2 | 21,099 | 7,144 |
| PC3 | 44,895 | 6,617 |
| PCBIO1 | 77,620 | 21,420 |
| PCBIO2 | 47,138 | 20,041 |
| PCBIO3 | 51,508 | 25,534 |
| Total | 726,661 | 170,237 |
