# Supplementary figures and images for: Amino Acids Hydrolyzed from Animal Carcasses Are a Good Additive for the Production of Bio-organic Fertilizer
Source: Front Microbiol. 2016 Aug 15;7:1290. doi: 10.3389/fmicb.2016.01290 (PMC4983570; doi:10.3389/fmicb.2016.01290)

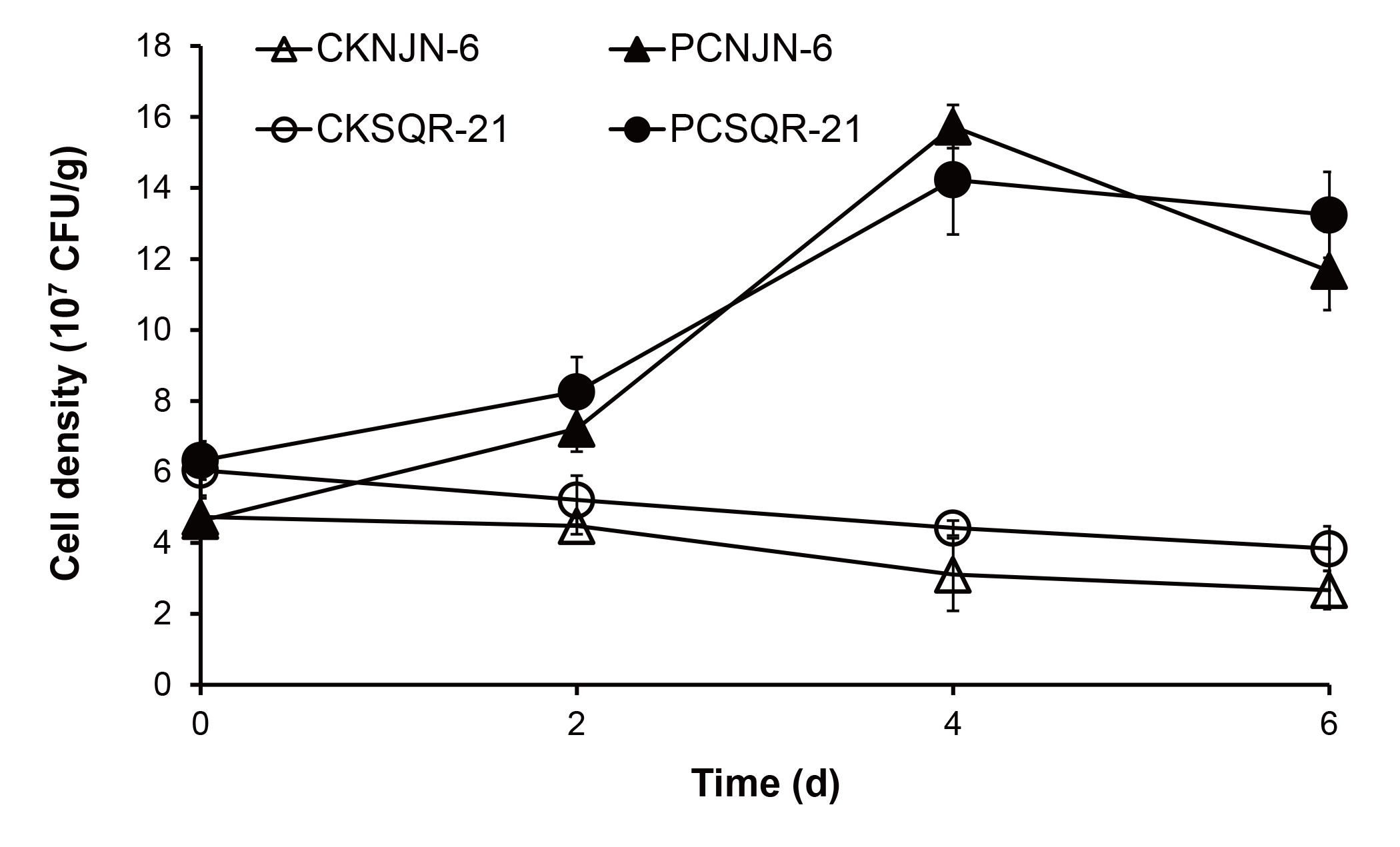

Supplement: FIGURE S1 — Effects of the novel solid-state fermentation (SSF) on the cell density variations of plant growth-promoting rhizobacteria (PGPR) NJN-6 and SQR21-gfp. [file Image_1.TIF]

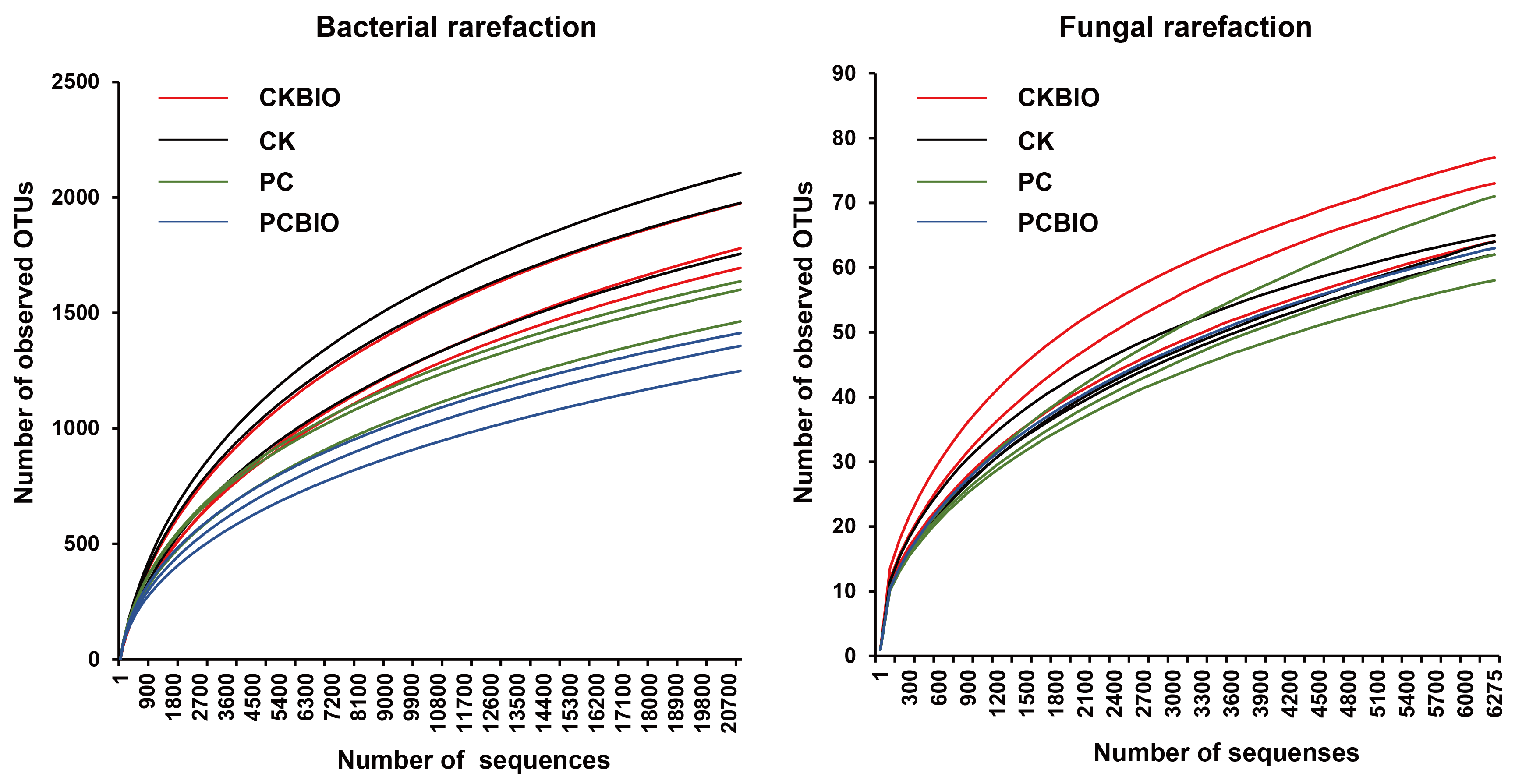

Supplement: FIGURE S2 — Rarefaction curves of bacterial 16S rRNA genes (A) and fungal ITS sequences (B) at 97% similarity levels of the different treatments and control. CK: the mature chicken manure compost; CKBIO: the mature chicken manure compost with strain SQR9 inoculation; PC: pre-compost of mixture piles of mature chicken manure and CLAA; PCBIO: inoculation of strain SQR9 in mixture piles of mature chicken manure and CLAA after pre-compost. [file Image_2.TIF]
